# Drivers of improved health sector performance in Rwanda: a qualitative view from within

**DOI:** 10.1186/s12913-016-1351-4

**Published:** 2016-04-08

**Authors:** Felix Sayinzoga, Leon Bijlmakers

**Affiliations:** Ministry of Health, Maternal & Child Health Department, PO Box 84, Kigali, Rwanda; Radboud University Medical Centre, Radboud Institute for Health Sciences, PO Box 9101, 6500 HB Nijmegen, The Netherlands

**Keywords:** Rwanda, Health system building blocks, Sector performance, Governance, District health, Web-based qualitative study

## Abstract

**Background:**

Rwanda has achieved great improvements in several key health indicators, including maternal mortality and other health outcomes. This raises the question: what has made this possible, and what makes Rwanda so unique?

**Methods:**

We describe the results of a web-based survey among district health managers in Rwanda who gave their personal opinions on the factors that drive performance in the health sector, in particular those that determine maternal health service coverage and outcomes. The questionnaire covered the six health systems building blocks that make up the WHO framework for health systems analysis, and two additional clusters of factors that are not directly covered by the framework: community health and determinants beyond the health sector.

**Results:**

Community health workers and health insurance come out as factors that are considered to have contributed most to Rwanda’s remarkable achievements in the past decade. The results also indicate the importance of other health system features, such as managerial skills and the culture of continuous monitoring of key indicators. In addition, there are factors beyond the health sector per se, such as the widespread determination of people to increase performance and achieve targets. This determination appears multi-levelled and influenced by both intrinsic and extrinsic motivation.

**Conclusion:**

It is the comprehensiveness and combination of interventions that drive performance in Rwanda, rather than a single health systems strengthening intervention or a set of interventions that target a specific disease. There is need for policy makers and scholars to acknowledge the complexity of health systems, and the fact that they are dynamic and influenced by society’s fabric, including the overall culture of performance management in the public sector. Rwanda’s robust model is difficult to replicate and fast-tracking elsewhere in the world of some of the interventions that form part of its success will require a holistic approach.

**Electronic supplementary material:**

The online version of this article (doi:10.1186/s12913-016-1351-4) contains supplementary material, which is available to authorized users.

## Background

Over the past 5–10 years, Rwanda has seen great improvements in several key health indicators, including most health outcomes in the domain of maternal health. Maternal mortality decreased significantly: the 2010 Demographic and Health Survey (DHS) estimated Rwanda’s maternal mortality ratio (MMR) at 476 deaths per 100,000 live births, down from 1071 deaths per 100,000 in the year 2000 [1]. The 2012 report of the Countdown to 2015 Collaboration ranked Rwanda as the country with the highest average annual rate of maternal death reduction, at 9 % [2]. Recent estimates by several UN agencies and the World Bank categorised Rwanda among 11 countries that are ‘on track’ to achieve target 5A of the Millennium Development Goals, which involves a decline of the MMR by at least 75 % between 1990 and 2013 [3].

These achievements are often attributed to a combination of improved population coverage and improved health service quality. In terms of coverage of maternal health services, the proportion of institutional deliveries is increasing (69 % according to the 2010 DHS; 90 % in 2013 according to the national health management information system [4]), while 98 % of pregnant women attend antenatal clinics at least once during their pregnancies [1]. The proportion of women who have their first antenatal consultation (ANC) during the first trimester of their pregnancy, though, was only 41 % in 2013, while only 31 % attended ANC at least four times before delivery [4].^1^ Other areas in which Rwanda achieved good progress include the national human Papillomavirus vaccination programme for the prevention of cervical cancer [5], the provision of antiretroviral therapy to pregnant and breastfeeding women who are HIV positive [6], and the national malaria control programme.

While improvements in service quality are much less reported in the literature, data from the national health management information system (HMIS) indicate substantial increases in the past few years in the percentages of pregnant women tested for anaemia, taking iron supplements to prevent anaemia, receiving tetanus toxoid immunisations, and the percentages of women detected with high risk pregnancies and those detected with (pre)eclampsia who are treated with magnesium sulphate [4]. Despite all these achievements, there is also evidence of deficiencies in Rwanda’s health system; for instance in the delivery of emergency and essential surgical services, particularly at district hospitals, which underlines the scope to further improve maternal and neonatal health [7].

Much has been said and written about Rwanda’s remarkable achievements in the domain of health. Several papers have appeared in prestigious international journals. Binagwaho et al. [8], Farmer et al. [9], Bucagu et al. [10] and Logie et al. [11] link the improved performance to governance—including donor coordination and the alignment of external aid to government policy—as well as to concrete initiatives such as community health insurance (*mutuelles de santé*) and performance-based financing (PBF). Other papers deal with particular features of the Rwanda health system, such as health research infrastructure [12], PBF [13] and community-based health insurance – not only in peer reviewed journals, but also in contributions to newsletters, weblogs and online communities of practice.

The central question that then arises is: *what makes Rwanda so unique?* Are the views of those who to a large extent control the knobs at the operational level of the country’s health system – i.e. the districts – any different from what has transpired so far in the international literature? We translated this into the following research question: what do Rwandan district health managers themselves consider to be the main drivers of improved health sector performance? And what scope do they see for further improvements?

It is the district health managers that have less voice, in conferences and in journal articles, yet they are the key agents who lead district health teams and supervise hospital staff so as to achieve targets and contribute to better overall health sector performance. It is important to note that district directors of health (DDH) and hospital directors (HD) in Rwanda have distinct roles and responsibilities. Both are members of the District Health Management Team (DHMT) and their positions are in principle equal in stature. The DDH, who is trained in public health and/or management, provides leadership, along with the Vice-Mayor in charge of social affairs of the district concerned, to the DHMT; while the HD is always a medical doctor and responsible for clinical matters. Both the DDH and the HD report to the Mayor, and through the Mayor to two different ministries: the Ministry of Health and the Ministry of Local Government. Mayors have performance contracts with the President that stipulate certain targets which are determined at the beginning of each fiscal year and reviewed periodically. Similarly, Ministry of Health officers as well as health staff employed at hospitals and health centres, all have their own performance contracts, with salaries that are partly fixed and partly variable, depending on their performance.

## Methods

In August-September 2014, we administered a web-based survey among district directors of health and district hospital directors to solicit their opinions and experiences. We invited all 30 district directors of health and all 42 district hospital directors in Rwanda, through a personal email, to participate in a web-based survey. The invitation contained a brief description of the purpose of the study and a unique hyperlink, which gave the invited persons direct internet access to the survey questionnaire. We used LimeSurvey, which is an open source survey application [https://www.limesurvey.com/], that allows respondents to save their responses at any given moment and, if desired, to resume completion of the questionnaire at a later point of time. The software allows researchers to monitor progress in the number of completed surveys and send customised email reminders to those who have not yet responded. No incentives were offered to participate, other than that we promised participants they would receive a summary of the findings as a token of our appreciation.

In designing part I of the questionnaire we distinguished between nine clusters of health system factors: they comprise the six building blocks, as defined by WHO [14], complemented with community health and intersectoral collaboration. The latter two have been cited in critiques of the WHO framework, which is on the one hand considered incomplete and too static, and on the other hand does not sufficiently take into account the interaction between a health system and the wider environment in which it operates [15–17]. We further divided the WHO building block infrastructure & supplies into two: physical infrastructure and medical technologies & supplies. The survey was in English and consisted of five parts, as shown in Table 1 (the full questionnaire is available at the link provided in Additional file 1).

**Table 1:** The five parts of the questionnaire with corresponding number of questions asked

I.	Drivers of performance *within* the health system in Rwanda	38 questions, covering 9 clusters
II.	Drivers of performance beyond the health system	15 questions
III.	Particular reasons why the performance in your district (or district hospital) with respect to maternal health may be different from the national average in Rwanda	4 statements
IV.	Personal viewpoints on health systems strengthening	6 open-ended questions
V.	Some personal background information	13 items, a combination of closed and open-ended questions

The first three parts contained questions and statements with Likert-type scales, ranging from 1 (not important at all) to 5 (very important) for questions; and for statements from 1 (strongly disagree) to 5 (strongly agree). All questions in parts I to IV had a provision to add free text and respondents were encouraged to explain their answers – in either English or in French – particularly for factors and statements about which they held strong opinions (scores 1 and 5).

No sampling was required: the directors of health of all 30 districts in Rwanda were invited as well as the directors of 42 district-level hospitals.^2^,^3^ We obtained their email addresses through the Ministry of Health. The email invitation to take part in the survey provided details about the purpose of the study and emphasised that all answers would be anonymised and treated confidentially. It was explicitly stated that by starting to complete the questionnaire participants consented to participate in the study.

Approval for the study was granted by the National Health Research Committee (NHRC; reference number NHRC/2015/PROT/006), and the Rwanda National Ethics Committee (RNEC; reference number 105/RNEC/2015).

## Results

### Response rate and background of participants

We obtained fully completed questionnaires from 24 Directors of Health and 33 Hospital Directors for a total of 57 respondents out of 72 persons targeted; which translates into a response rate of 79.2 %.^4^ Almost half of the respondents (47 %) had been in their positions (as DDH or HD) for more than four years (before 2010), while only three of them had been appointed earlier in the year (2014). In terms of years of professional experience, the two groups of respondents were very similar: 10 years on average in both group, ranging from four years to 26 and 24 years, for DDH and HD respectively. DDH were 39 years of age on average, HD were slightly older (41 years). Only one HD out of 33 who participated in the survey was female, compared to nine females out of 24 DDH (38 %).

All of the hospital directors had a medical background: 16 held a master degree (MPH, M-Med or M-Epid), ten held a bachelor’s degree and one a PhD (missing information for the remaining six). Among the DDH, there were 12 bachelors (of which seven public health, four A-0 license and one other), outnumbering nine others who held a master title (six MPH, three other), with three missing data.

### Drivers of performance within the health sector itself

Respondents were asked to give their personal judgement (on a scale of 1 to 5) about the extent to which a series of 38 health system factors have contributed to Rwanda’s improved health sector performance. The top five factors that received the highest scores are listed in Table 2. The five health systems factors considered to have contributed least are listed as well.

**Table 2:** Average scores assigned by respondents (*N* = 57) to *other* drivers of performance, beyond the health system, ranked in order of importance

Rank	Factor	Average score^a^	Range
1	Determination by central government to build a better society	4.5	3 to 5
2	Determination among local govt. administrators to build a better society	4.3	2 to 5
3	Increased awareness among population about health risks	4.3	2 to 5
4	Improved water & sanitation and hygienic conditions	4.3	3 to 5
5	Improved literacy levels, particularly among women	4.2	2 to 5
6	Increased child spacing and family planning; lower fertility levels	4.2	2 to 5
7	Better individual behaviour and protection against health hazards	4.2	2 to 5
7	More focus of local leaders and programme managers on vulnerable groups	4.2	1 to 5
9	Improved economic conditions of Rwandan households	4.1	2 to 5
10	Stronger collective effort of the population to build a better society	4.1	3 to 5
11	More external support from donors and international agencies	4.1	2 to 5
12	Increased population awareness about rights and duties	4.1	1 to 5
13	Determination among non-state actors to build a better society	4.0	2 to 5
14	Improved diets, better nutritional status	4.0	2 to 5
15	Increased sense of responsibility of people to manage their own lives	4.0	2 to 5

^a^Scoring on a scale from 1 (not important at all) to 5 (very important)

**Fig. 1:**
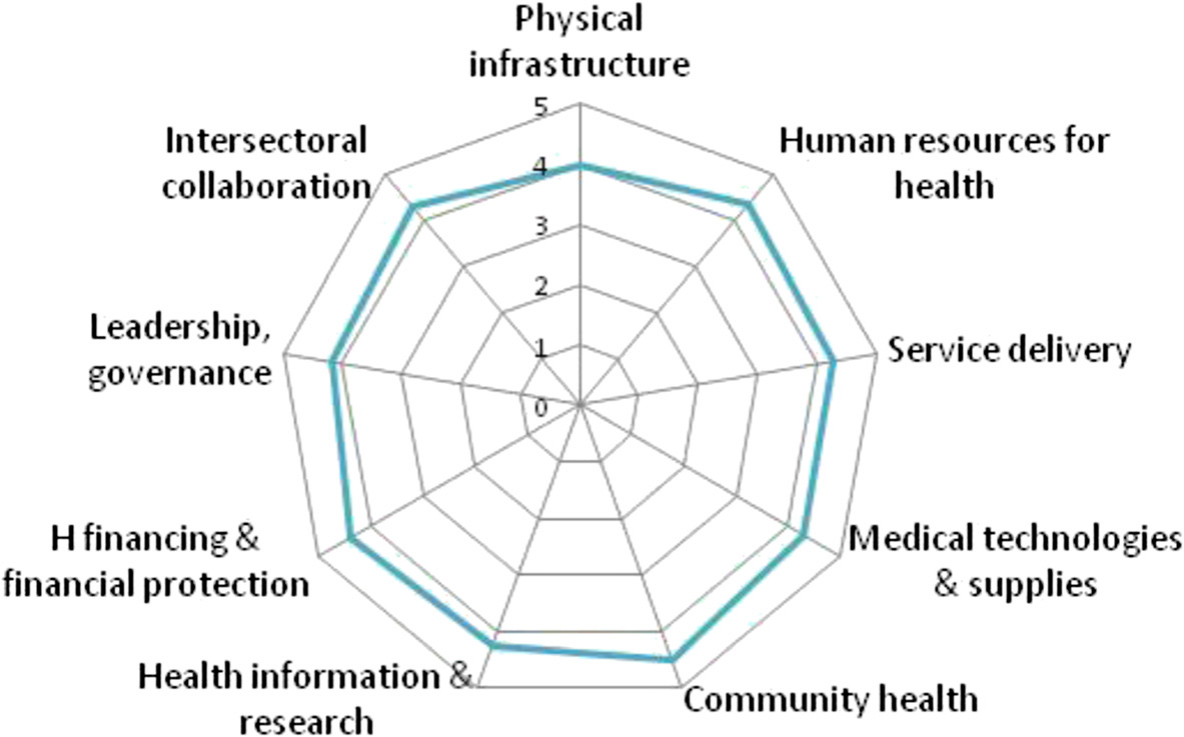
Average scores assigned by respondents (*N* = 57) to nine clusters of health system drivers of performance

Figure 1 illustrates the relative importance of nine clusters of health system factors, as per the judgement of the respondents. Community health activities came out as having contributed most to better health sector performance, followed by improvements in human resources for health. Expansion of physical health infrastructure scored lowest, but it is to be noted that the differences in average scores between the nine clusters are limited, with average scores ranging from 4.0 to 4.5.

Box 1 Top five features in which the Rwanda health system distinguishes itself from health systems in other countries, according to respondents (*N* = 57)

**Table:** 

1. Leadership (22 respondents) 2. Community health insurance/*mutuelles de santé* (18 respondents) 3. Community health/CHW (15 respondents) 4. Focus on vulnerable groups, including mothers and children, and equitable access (9 respondents) 5. Performance monitoring and accountability (9 respondents)	

In narrative comments that respondents provided to explain their scores, some argued explicitly that it is the combination of factors that has led to success. One respondent expressed it as follows:

*“It’s our health system as a whole, with the various activities that we undertake, the way the system is organised and our overall policy and leadership; it isn’t for just one reason that we manage to improve our health indicators.”*

### Other drivers of performance, beyond the health sector

Respondents were also asked to give their personal judgement about the importance of *other* factors, not limited to the health sector per se. Table 3 presents averages of the scores obtained for 15 such factors, ranked from the highest average score to the lowest. Here again, the differences are quite small.

**Table 3:** Factors considered to have contributed most and least to Rwanda’s improved health sector performance

Top five factors:
1. Widespread presence of community health workers
2. Expansion of the service package covered by community-based health insurance
3. Increase in the density of health centres country-wide
4. Improved diagnostic methods (laboratory investigations, rapid tests, radiology) at various levels of the health system
5. Improved patient referral system.
Bottom five factors:
1. Increase in private health facilities country-wide
2. Improved specialist services at district hospitals
3. Health research
4 Health legislation and enforcement
5. Improved technologies for medical treatment.

The central government’s determination to build a better society is perceived as the main driver of health sector performance, followed by local governments’ determination to build a better society. This is illustrated by the following quotes from respondents:

*“When central government is committed, any target can be achieved.”*

*“Local leaders are key in health improvement.”*

*“Performance contracts have made a big difference.”*

Improved water supply & sanitation are also considered important determinants, along with several individual cognitive-behavioural factors, such as the general public’s increased awareness of health risks, improved literacy levels (in particular among women), increased child spacing (lower levels of fertility), and more conducive individual behaviour and personal protection against health hazards.

We list some illustrative quotes from respondents:

*“In the past, community behaviours contributed much to preventable illnesses, but now people have changed their dietary pattern and they are protecting themselves against potential risks”*

*“Mass campaigns and frequent health education have increased awareness among the general population about health issues.”*

*“Literacy levels, particularly among women, have improved as shown by the 2012 census report of our National Institute of Statistics.”*

*“We have seen an impressive progress in population health in* < name of district>*, and this has been boosted by child spacing, even though our fertility rate is still higher than the national average.”*

*“People in our district take more responsibility for their own lives, e.g. ensuring personal hygiene, motorcyclists wearing helmets.”*

Collective efforts, with specific objectives or aimed at certain target groups, were recognised as well:

*“District administrators try to identify and assist vulnerable people at the district hospital, while sector administrators help vulnerable people to access health services at health centres.”*

*“There many examples of ways in which we make collective efforts to build a better society: e.g. through the* Agaciro *development fund,*^5^*the* Ndi Umunyarwanda *scheme,*^6^*the* Umuganda *community service.*^7^*”*

An interesting divergence of opinions emerged about the role of external aid in achieving health sector improvements, as illustrated by the following two quotes:

*“External aid has been of great added value and has helped to maintain momentum in health, water and sanitation issues; development partners are increasingly aligning their interventions to government priorities.“*

*“Foreign aid has not been so important.”*

### Deviations of district health performance from the national average, and reasons given

Respondents were asked how their districts performed in the area of maternal health compared to the national average in terms of service coverage, service quality and health outcomes, in particular maternal mortality.

- More than half of the respondents (57 %) said their districts performed better than the national average on service coverage indicators, such as % of assisted hospital deliveries and % of women attending antenatal consultation (ANC); a quarter (26 %) believed their district’s performance was below the national average; while the remaining 17 % indicated it was similar or they were not sufficiently familiar with their districts’ performance in order to judge. The two factors mentioned most as reasons for high coverage indicators are:
  1. Good collaboration between district/sector administration, community health workers (CHW) and health facility staff; and, related to that,
  2. Strong commitment of CHW to register all pregnant women and encourage them to attend ANC at an early stage.

Community sensitisation and higher levels of awareness in general were also mentioned, without referring to any of the actors involved. Several of the respondents who indicated their coverage rates were relatively low, attributed this to the low rates of enrolment in community based health insurance in their districts.

- With regard to service quality, respondents were asked to comment on the following statement: ’My district’s (or district hospital’s) performance on service *quality* indicators – such as % of pregnant women tested for anaemia, the % of pregnant women taking iron supplements to prevent anaemia, the % of women receiving tetanus-toxoid immunisations – is better than the national average for Rwanda.’

This time, more than two-thirds (70 %) claimed their districts performed better than the national average. Some specifically singled out the contribution of CHW to achieving good results. Among those who disagreed with the statement (15 %), some admitted that the health facilities in their districts were not doing enough to systematically provide the full range of antenatal care services, mostly because of shortages of trained staff and/or medical supplies.

- Almost two-thirds of the respondents (63 %) reacted affirmatively to the following statement on health outcomes: ‘My district’s (or district hospital’s) performance on maternal health outcomes – in particular maternal mortality – is better than the national average for Rwanda.’ Others referred to the much improved patient referral system, with ambulances based at certain remote health centres, mainly to facilitate obstetric emergency referrals. Most of those who neither agreed nor disagreed with the statement (16 %) indicated they would not want to speculate about maternal mortality rates, with some looking forward to seeing next year’s DHS results. Among those who disagreed (22 %), hence indicating their health outcomes were worse than the national average, one respondent pointed to the tendency of pregnant women in his district to register late for ANC, or to come to the hospital at a late stage in case of complications during delivery; another one referred to the widespread belief in traditional medicine which would deter certain people from using modern health facilities.

Two-thirds of the respondents (68 %) were generally optimistic about the possibilities to achieve good maternal health results in their districts, compared to places elsewhere in Rwanda, with many of them referring to a very high level of commitment among health staff and strong leadership; for instance:

*“We have a strong willingness among all stakeholders, including politicians, to work together and increase our performance.”*

Others pointed at the presence of well-trained staff, the proximity of health facilities that provide basic obstetric care and the availability of ambulances to take care of obstetric emergencies. Some respondents were very adamant in their statements:

*“We have everything that enables us to achieve good maternal health results.”*

*“There is no special reason why we can’t achieve good results; we have to work hard”.*

### Personal viewpoints on health systems strengthening

Asked for examples from their own experience that best illustrate Rwanda’s good achievements in the health sector over the past five years, almost half of the respondents (42 %) referred to declines in mortality rates, in particular maternal mortality, but also neonatal, infant and child mortality. Others mentioned declines in the incidence and/or mortality from infectious diseases (malaria, HIV), the increase in assisted deliveries or the use of family planning methods.

Improvement in access to health services was mentioned by 30 % of the respondents, where some referred to physical infrastructure, but the majority to the high coverage of community health insurance. Not less than 37 % pointed to Government political commitment, the strong leadership and governance issues (improved policies and guidelines) as factors that had made it possible to make progress. One person emphasised the very close monitoring at all levels of trends in key indicators and the corrective measures that were being taken if targets were not attained:

*“It’s controlled every day”.*

Community health workers (CHW) and increased levels of community participation were mentioned by a quarter (26 %). With regard to human resources for health (23 %), some emphasised the increased numbers of trained professionals, while others referred to higher levels of technical capacity, motivation and commitment. Still others singled out specific health system improvements, such as the community health information system (SISCOM) and rapid SMS alerts; or broader factors, such as increased budget allocations to health and improved resource management.

### Lessons learned and further learning needs

When asked what was the most important thing they had learned in their professional careers about health systems strengthening, 58 % of the respondents mentioned governance or governance related topics, such as leadership and political commitment at the highest level, a clear organisational/ institutional framework and coordination, strategic/target-based planning, accountability, team work and stakeholder engagement, including involvement of mid-level health care providers and community health workers.

The second most frequently mentioned health systems feature that respondents cited as something they had learned in their professional careers was health information and the use of data for informed decision making (14 %).

The favourite areas in which respondents said they would personally like to learn more are monitoring & evaluation and HMIS (16 %), followed by research (11 %). Others would like to learn more about health planning & management, hospital administration, health financing, health insurance, human resource management, quality assurance, innovation, e-health. Some respondents (16 %) wanted to improve their clinical competence (as opposed to managerial skills), and this was mostly in the domain of maternal & child health or emergency obstetric care.

### Barriers and proposed changes

Respondents were then asked to mention, from their own personal perspective, the two most important barriers to further improvements in health sector performance in Rwanda. Human resource related barriers were mentioned most frequently (60 %): mostly staff shortages and the high rates of attrition. Some mentioned specific cadres of which there were shortages: medical doctors, nurses, specialists, laboratory technicians. Other barriers that were mentioned frequently are: general poverty and ignorance on the side of the community; the funding gap in the health sector, with some saying this posed challenges for the procurement and maintenance of medical equipment; the low rates of adhesion to community-based health insurance; and inadequate physical infrastructure.

When asked to mention ’the one thing you would like to see changed in the current set-up of the Rwandan health system’, 13 respondents (23 %) referred to human resources for health. Some wanted the number of trained professionals to be further increased, others suggested measures to reduce staff turnover and/or help retain staff. Still others wanted better or more equitable employment conditions, non-monetary incentives for health staff, inclusion of district health managers in PBF, and protection of service providers from litigation. Only one person expressed discontent, by saying he did not like

*“… the manner in which some of the higher level health authorities treat their personnel”*.

Some respondents suggested structural changes in decentralisation or in coordination among actors: between the Ministry of Health headquarters and districts, or between the Ministry and the Rwanda Biomedical Centre. Others wished for more realistic planning; improvements in the patient referral and counter-referral system; and better information to the general public, not only about their entitlements, but also about their own responsibilities and obligations as far as health is concerned.

## Discussion

District health managers consider a wide variety of health system features and factors as reasons for the recent successes in Rwanda’s health sector. While some elements had somewhat higher scores than others, the differences were actually quite small, and it appears to be the complementarity of various interventions and sub-systems that is important. Respondents mention the widespread presence of community health workers (CHW) and health insurance as the main factors that have led to Rwanda’s improved health sector performance. Rwanda does have a dense network of CHWs who deliver a broad range of preventive and curative services in their own communities. For every 800-1000 people in Rwanda, there is one maternal health CHW who monitors pregnant women and their new-borns, and two multi-disciplinary CHWs (*binômes*) who carry out integrated community case management, malnutrition screening, and other preventive and behaviour change activities [18]. The Ministry of Health has also established a standardized community health information system (SISCOM) which makes data collected by CHWs available at the (sub-)national levels and which complements the health facility-based data in the national health information system. Enrolment in community based health insurance is very high, with 90.6 % of the population that was enrolled as of June 2012, with another 7 % covered by civil service, military or private insurance schemes [9]. Many preventive interventions, such as bed nets and vaccinations, are fully covered by the insurance package, along with treatment for HIV, tuberculosis and some cancers. Apart from the annual premiums, subscribers pay 10 % co-payments at the point of care for services that are not fully covered. Poor people pay smaller premiums.

Our results further indicate the importance of other health system features, such as improved managerial skills and a monitoring & evaluation culture nurtured by a widespread and multi-levelled determination to increase performance, which is not solely driven by individuals’ financial interest.

PBF, which was adopted as a nationwide strategy in 2005, rewards community health worker cooperatives, health centres, and district hospitals for improved patient follow-up and certain primary care indicators, such as the proportion of women delivering at health facilities and children completing the full course of immunisations. Somewhat to our surprise, PBF was mentioned less as one of the key drivers of performance than we had expected.

This survey is unique in the sense that it is the first of its kind to interrogate district health managers about factors that drive performance. They are of the opinion that it is a multitude of factors, inside and outside the health sector, that have determined Rwanda’s steep progress towards achieving universal health coverage and meeting most of the Millennium Development Goals by 2015.

### Limitations

The survey has three methodological limitations. Firstly, a validated research instrument was not available, and we therefore designed the questionnaire based on what is reported in the literature about the complexity and dynamics of health systems and determinants of health sector performance. The possibility for respondents to explain their responses in narrative form, especially their scores on Likert scales, made it possible for us to analyse to some extent how questions were actually understood.

Secondly, the survey did not make use of any comparator group or situation, of which the internal validity might have benefited. The choice to direct the survey to two different types of respondents (district directors of health and hospital directors) was not with the intention to compare them.

Third, although the survey was web-based, it did not allow any interaction among the respondents, or between researchers and respondents. The questions were primarily close-ended, with ample opportunity for participants to explain their responses, but some indicated that their writing skills (in English or French) limited them in expressing themselves.

The survey’s external validity is not much of a problem: at 79 %, the response rate was very high by international standards for web-based surveys [19] and also much higher than the response rates usually found in postal surveys [20–22]. We were proven right in our *a priori* assumption that in Rwanda, which has a good information technology infrastructure and a culture in which civil servants feel obliged to participate in initiatives coming from the central government, a web-based survey among health professionals should be feasible. This is not to be taken for granted though in other countries in Sub-Saharan Africa. We found no indication that the non-respondents – 15 in total – might be different in any way from those who participated in the survey.

### Attribution

Several authors have tried to link Rwanda’s successes in health to specific initiatives, but attribution remains tricky.

- Bucagu et al. [10] reviewed evidence of the impact of health systems strengthening on coverage of maternal health services in Rwanda. In their description of health sector reforms, they identified the year 2006 as the point in time when three important policies were scaled up: facility-based childbirth (assisted deliveries), performance-based financing (PBF) and community-based health insurance (CBHI). From their analysis of trends in service coverage indicators over two periods (2001–2005 and 2006–2010), they identified four main factors that drove changes in four different indicators of maternity care coverage: health workforce, PBF, CBHI and leadership & governance. The relative weight of each of these factors could not be established, and it is not quite clear what the possible contribution was of other factors, such as CHW and health information management for informed decision making, or whether they were considered at all.
- Basinga et al. [13] assessed the effect of performance-based payment of health care providers on the use and quality of child and maternal care services in health care facilities in Rwanda. They conducted a survey of 166 facilities, half of which were randomly assigned to begin pay-for-performance (P4P) funding in 2006, with the other half continuing the traditional input-based funding, for a period of almost two years. The P4P scheme turned out to have had the greatest effect on those services that had the highest payment rates and needed the least effort from the service provider. The authors concluded that financial performance incentives can indeed improve both the use and quality of maternal and child health services.
- Farmer et al. [9] observed a certain disagreement among scholars and opinion leaders globally about the reasons for Rwanda’s success. Analysing the country’s quest to rebuild the health system after the 1994 genocide, they identified lessons learned in relation to six key factors: National leadership, Health systems approach, Country ownership, Community-based care, Evidence-based policy making, and Cross-sector collaboration. The health systems approach remains loosely defined, and typically misses out on health service delivery.
- The pivotal role of CHW’s, and especially the routine utilisation of CHW data to ensure performance monitoring and quality assurance was highlighted by Mitsunaga et al. [18]. This ties in well with an attribute of Rwanda’s health system that has not received much attention in the literature, namely self-assessment and peer evaluation.

Our survey confirms an observation reported recently by Janssen et al. [23] that health workers themselves reviewing statistics and monitoring adherence to guidelines, both for clinical work and managerial tasks, is characteristic for Rwanda and an important aspect of the overall culture of public sector performance management. It received a boost with the adoption and expansion of PBF, and it thereby offered opportunities for shared learning and continuous improvement of performance. PBF should therefore not be simply seen as a financing mechanism, or an initiative that enhances staff motivation and/or community involvement, but also as an instrument that nurtures a climate of continuous performance appraisal and problem solving. Rwanda has the conditions in place for PBF to play this role.

### Uniqueness of Rwanda

The uniqueness of the Rwanda model is three-fold. Firstly, the health system has been built around the notion that it is not a single health systems strengthening intervention, or a focus on a particular condition or a disease that drives performance [24], but rather the comprehensiveness and combination of interventions that complement and reinforce each other, particularly in maternal health. Secondly, there is strong political commitment in Rwanda – with the national leadership, district health managers and local government administrators cognisant of their interdependence – to improve maternal health, and health more in general. Combined with this, the central government has put in place mechanisms of close oversight and control of the performance of health institutions and individual health workers, primarily through performance contracts that stipulate certain financial rewards and punitive measures. This means that staff motivation is to some extent extrinsic. Added to this, there is a strong sense in Rwanda that factors beyond the health sector – such as literacy, nutrition and water & sanitation – cannot be ignored. Such strong political will has been called for globally for a long time, and its importance continues to be reemphasised, both in the context of general health systems strengthening [25] and that of maternal health specifically [26]. And thirdly, there is widespread cognisance of the fact that the fabric of society has an important role to play in achieving better health. In particular the strong involvement of women in collective health actions within local communities around reproductive health and safe delivery – often in close collaboration with local health workers – is remarkable. Internationally, the latter type of action is increasingly being emphasised as a *sine qua non* for improving maternal health [27].

It will be interesting to see the results of the 2015 Demographic and Health survey, which might be expected to confirm the health impact of Rwanda’s multi-faceted approach to achieving its health and development goals.

## Conclusion

The present study has elicited how Rwanda has translated its policy intentions into a set of comprehensive and complementary actions embedded in a culture of performance management that are meant to strengthen the health system; and which have actually resulted in a steep increase in performance. In the meantime, there is need for policy makers and scholars to acknowledge the complexity of health systems, the interdependency of what is often referred to as ‘health system building blocks’ and the overall culture of performance management in the public sector. It calls for more holistic analyses and a tuning down of the high expectations from single interventions and from randomised controlled trial designs as the most powerful type of study. One of the big pitfalls for policy makers is to ignore local context and complexity when trying to replicate or fast-track the scaling-up of promising trial results and pilot projects. As much as the Rwanda experience has a lot to offer as a model that appears robust, it will be difficult to replicate it elsewhere in the world unless the bigger picture is taken into account.

## Additional file

Additional file 1: LimeSurvey questionnaire. (PDF 191 kb)

## Abbreviations

ANC: antenatal care
CBHI: community-based health insurance
CHW: community health worker
DDH: district director of health
DHMT: district health management team
DHS: demographic and health survey
HD: hospital director
HMIS: health management information system
MMR: maternal mortality ratio
P4P: pay-for-performance
PBF: performance-based financing

## Footnotes

1 Recent HMIS data however show a reduction of ANC coverage between 2011 and 2012, especially for those who attend ANC clinics during the first quarter of their pregnancies and those who have a minimum of four visits. This could be due to underreporting of ANC visits at national referral hospitals and/or private health facilities; or to an inaccurate projection of expected pregnancies (4.1 % of total population), which does not take into consideration the rapid adoption of family planning services.

2 Some of the larger districts have more than one hospital.

3 The directors of the five national-level referral hospitals were not included in the survey: the university hospitals in Kigali and Butare, King Faysal hospital in Kigali, the neuro-psychiatric hospital in Ndera and the Rwanda military hospital.

4 Response rate 80.0 % among DDH, 78.6 % among hospital directors; difference not statistically significant.

5 A Solidarity Fund, launched by the Rwanda Government in 2012, to which citizens and friends of Rwanda can donate money. ‘Agaciro’ is a Kinyarwandan word that can best be translated as ‘dignity’.

6 A Government initiative launched in 2013 aiming at reconciling Rwandans ahead of the 20th anniversary of the genocide in 2014. ‘Ndi Umunyarwanda’ means ‘I am Rwandan’.

7 Mandatory day of community service, the last Saturday of each month. ‘Umuganda’ means ‘coming together for a common purpose’.
