# Supplementary material for: Drivers of improved health sector performance in Rwanda: a qualitative view from within
Source: BMC Health Serv Res. 2016 Apr 8;16:123. doi: 10.1186/s12913-016-1351-4 (PMC4826525; doi:10.1186/s12913-016-1351-4)
Supplement: Additional file 1: — LimeSurvey questionnaire. (PDF 191 kb) [file 12913_2016_1351_MOESM1_ESM.pdf]

# LimeSurvey questionnaire

# 'Factors driving performance in the health sector in Rwanda'

## Introduction

Over the past 5-10 years, Rwanda has seen great improvements in several key health indicators. In the domain of maternal health, for instance:

- Many health outcomes have improved, such as maternal mortality: the 2010 DHS estimated Rwanda's maternal mortality ratio at 476 deaths per 100,000 live births, down from 1,071 deaths per 100,000 in the year 2000; the 2012 report of the Countdown to 2015 collaboration ranked Rwanda as the country with the highest average annual rate of maternal death reduction, at 9%.[1]
- Health service coverage has improved, such as institutional deliveries (69% according to the 2010 DHS; 87.6% according to the national HMIS) and antenatal visits (98% of pregnant women attended antenatal clinics at least once during their pregnancies, according to the 2010 DHS).[2]
- Service quality has improved as well, as indicated by improvements in the % of pregnant women tested for anaemia, the % of pregnant women taking iron supplements to prevent anaemia, the % of women receiving TT immunisations, the % of women with (pre)eclampsia treated with magnesium sulphate, the % of women detected with high risk pregnancies.

A lot has been said and written on Rwanda's remarkable achievements in the domain of health (not just in maternal health). Several papers have appeared in prestigious international journals, for instance Bucagu et al. (2012)[3], Basinga et al. (2011)[4], Binagwaho et al. (2014)[5].

The present survey tries to solicit your views, as a district director of health, or as a district hospital director, on a number of issues, so as to corroborate some of the claims that have been made by policy makers, practitioners, scientists and representatives from international development agencies. Please note that we will anonymise all survey results. It means that your name will not be disclosed to anyone. And the names of districts and hospitals will be not be mentioned in any report, so that readers will not be able to trace back who said what. You should therefore feel free to express your honest personal opinion, even if it may make things look not so good; or if in your view reality on the ground is less positive than the official policy.

[1] Countdown to 2015 Maternal, Newborn & Child Survival: http://www.countdown2015mnch.org/

[2] Recent HMIS data however show a reduction of ANC coverage between 2011 and 2012, especially for those who attend ANC clinics during the first quarter of their pregnancies and those who have a minimum of four visits. This could be due to underreporting of ANC visits at national referral hospitals and/or private health facilities; or to an inaccurate projection of expected pregnancies (4.1% of total population), which does not take into consideration the rapid adoption of family planning services.

[3] Bucagu M, Kagubare JM, Basinga P, Ngabo F, Timmons BK, Lee AC. Impact of health systems strengthening on coverage of maternal health services in Rwanda, 2000-2010: a systematic review. *Reproductive health matters*. 2012;20(39):50-61. Epub 2012/07/14.

[4] Basinga P, Gertler PJ, Binagwaho A, Soucat AL, Sturdy J, Vermeersch CM. Effect on maternal and child health services in Rwanda of payment to primary health-care providers for performance: an impact evaluation. *Lancet* 2011;377(9775):1421-8. Epub 2011/04/26.

[5] Binagwaho A, Farmer P, Nsanzimana S et al. Rwanda 20 years on: investing in life. *Lancet* 2014; 384(9940): 371-375.

This survey is part of a research project that examines health sector responsiveness to maternal health issues and concerns in Rwanda and South Africa. The Ministry of Health in Rwanda is one of the participating partners in this project, along with academic institutions in South Africa and the Netherlands. For all questions you may have on this project, please contact Dr Felix Sayinzoga (at felix.sayinzoga@gmail.com) or Dr Fidèle Ngabo (at ngabog@gmail.com).

The survey consists of five parts:

I. Drivers of performance within the health system in Rwanda
II. Drivers of performance beyond the health system
III. Particular reasons why the performance in your district (or district hospital) with respect to maternal health may be different from the national average in Rwanda
IV. Your personal viewpoints on health systems strengthening
V. A bit of personal background.

It will probably take you 30 minutes, or even a bit more to complete this survey.

It is important that you complete the whole questionnaire. Please do not leave anything blank, because we would not know what that means. In case you are not sure, just write "Not sure" or "Don't know". In case a question does not apply, please write "Not applicable" or "NA".

We do take interest in explanations that you may be able to provide. That is why we encourage you to provide comments, where appropriate.

**Part I: Drivers of performance**

*We would like you to give your personal judgement about a number of health system factors that are often cited as 'drivers of performance'.*

*Please give a rating, on a scale from 1 to 5, to express* ***to what extent in your view each of the factors listed below have indeed contributed to improved health sector performance in Rwanda****.*

*Key:*

1 = not important at all 2 = not so important 3 = neither unimportant nor important
4 = important 5 = very important
In case you have no idea please give the rating 9.

*Please do provide brief comments (in your own words) or examples to justify your ratings, especially where they are very high (5) or very low (1). You may write in English or French, whatever you prefer.*

1) More health centres and clinics country-wide 1 – 2 – 3 – 4 – 5 – 9

Comment: ______________

2) More private dispensaries, clinics, hospitals 1 – 2 – 3 – 4 – 5 – 9

Comment: _______________

3) Increased health workforce (in terms of numbers of health staff) at health centre level

4) Increased health workforce (in terms of numbers of health staff) at district hospitals

5) Better trained health workers at health centre level

6) Better trained health workers at district hospitals

7) Expansion of the standard service package available at health centres

8) Improved standard treatment guidelines for various illnesses and conditions

9) Improved specialist services at district hospitals

10) Improved specialist services at referral hospitals

11) Improved patient referral system

12) Improved diagnostic methods (laboratory investigations, rapid tests, radiology) at various levels

13) Improved technologies for medical treatment

14) Improved availability of essential drugs and medical supplies

15) Widespread presence of community health workers (CHW: *binômes, animatrices de santé maternelle*)

16) Wider scope of activities undertaken by CHW

17) Improved quality of activities undertaken by CHW

18) Improved Community Health information system (SISCOM)

19) Better flow of data and information between the different layers of the health system (community, health centre, district hospital, referral hospital)

20) Closer monitoring of key HMIS indicators by district health management teams

21) More commitment among health professionals to achieve set targets

22) More commitment among health staff to reach out to particular vulnerable groups

23) More commitment among the Ministry of Health leadership to reduce disparities (regional disparities or between population subgroups)

24) Improved health research

25) Expansion of performance-based financing (PBF)

26) Higher population coverage (enrolment rates) of community-based health insurance (*mutuelles de santé*)

27) Expansion of the service package covered by community-based health insurance (*mutuelles de santé*)

28) Increased overall level of health sector funding

29) More clarity among health workers themselves (within their teams) about who is responsible for what and accountable to whom

30) More clarity from health administrators towards community members about what clients can expect from the services that hospitals and health centres provide

31) Obligations for health workers/administrators to respond to opinions, suggestions, or complaints from clients/community members.

32) A more comprehensive national health strategy, accompanied by periodic strategic reviews

33) More realistic district health planning and budgeting, compared to what used to be the case

34) Improved health legislation and law enforcement

35) Increased inter-sectoral collaboration at district level and below between the health sector and other sectors

36) Increased willingness of the Ministry of Health leadership to be held accountable for their decisions

37) Increased willingness of the Ministry of Health leadership to be held accountable for the performance of the health sector as a whole

38) Increased involvement of other ministries in health matters nation-wide.

<u>**Part II: Other drivers of performance, beyond the health sector**</u>

*Now we would like you to give your personal judgement about a number of <u>other factors</u> (not limited to the health sector per se) that are often cited as 'drivers of performance'. As above, please give your rating to express* ***to what extent <u>in your view</u> each of these factors have indeed contributed to improved health sector performance in Rwanda.***

*Key:*

1 = not important at all  2 = not so important  3 = neither unimportant nor important
4 = important  5 = very important
In case you have no idea please give the rating 9.

*Again, <u>please do provide comments</u> (in your own words) or examples to justify your ratings, especially where they are very high (5) or very low (1).*

1) Improved general economic conditions of Rwandan households, especially income
1 – 2 – 3 – 4 – 5  – 9

Comment: _____

2) Improved literacy levels, particularly among women

3) Increased child spacing and family planning; reduced levels of fertility

4) Improved water supply, environmental sanitation and conditions of hygiene

5) Improved diets, better nutritional status

6) Improved individual behaviour and personal protection against health hazards (mosquito bites, smoking, unhealthy food, lack of physical exercise)

7) Increased awareness among the general population about health issues and risks

8) Increased awareness among the general population about their rights and duties

9) Increased sense of responsibility among people to manage their own lives

10) Increased focus of local leaders and programme managers on vulnerable groups

11) Stronger collective effort of the population in general to build a better society.

12) More determination among the central government to build a better society

13) More determination among local government administrators to build a better society

14) More determination among non-state actors (civil society) to build a better society.

15) More external support from donors and international agencies to help Rwanda build a better society.

**Part III: Reasons for deviations, if any, in the performance of your own district (or district hospital) in respect of maternal health**

*On a scale of 1 to 5, please indicate whether you agree or disagree with the following statements*

1) My district's (or district hospital's) performance on service coverage indicators – such as % of hospital deliveries and % of women attending antenatal consultations – is better than the national average for Rwanda.

| 1 | 2 | 3 | 4 | 5 |
|---|---|---|---|---|
| Strongly disagree – | Disagree – | Neither agree nor disagree – | Agree – | Strongly agree |

9 = Don't know

The main reason for this is ____________

2) My district's (or district hospital's) performance on service quality indicators – such as % of pregnant women tested for anaemia, the % of pregnant women taking iron supplements to prevent anaemia, the % of women receiving TT immunisations – is better than the national average for Rwanda.

| 1 | 2 | 3 | 4 | 5 |
|---|---|---|---|---|
| Strongly disagree – | Disagree – | Neither agree nor disagree – | Agree – | Strongly agree |

9 = Don't know

The main reason for this is _____________

3) My district's (or district hospital's) performance on maternal health outcomes – in particular maternal mortality – is better than the national average for Rwanda.

| 1 | 2 | 3 | 4 | 5 |
|---|---|---|---|---|
| Strongly disagree – | Disagree – | Neither agree nor disagree – | Agree – | Strongly agree |

9 = Don't know

The main reason for this is _____________

4) In my district it is relatively difficult to achieve good maternal health results, compared to most other districts in Rwanda.

| 1 | 2 | 3 | 4 | 5 |
|---|---|---|---|---|
| Strongly disagree – | Disagree – | Neither agree nor disagree – | Agree – | Strongly agree |

9 = Don't know

The main reasons for this are

a. ___

b. ___

**Part IV: Viewpoints on health system strengthening**

*Please respond to the following open-ended questions. As before, you may respond in English or French, whatever you prefer.*

1) From my own personal experience as a professional, the following two examples show why the health sector in Rwanda has achieved a lot of progress over the past 5 years.

    a. Example 1: _____

    b. Example 2: _____

2) In my view, the two most important aspects in which the health system in Rwanda distinguishes itself from health systems in other countries are:

    a. First aspect: _____

    b. Second aspect: _____

3) The most important thing I have learned in my professional career about health system strengthening is: ______________

4) The one area in which I personally would like to learn more is: _________

5) In my personal view, the two most important barriers to further improvements in health sector performance in Rwanda are:

    a. Barrier 1: _____

    b. Barrier 2: _____

6) If there was one thing I would like to see changed in the current set-up of the Rwandan health system it is the following : _________

**Part V: Personal background**

*Please note that we will anonymise all survey results. It means that your name will not be disclosed to anyone. The names of districts and hospitals will be not be mentioned so that readers will not be able to trace back who said what. Nevertheless we need the following information from you.*

1. My present position is that of O Director of Health
   O Hospital director
   O Other (specify): ..........

2. I have held this position since ... (indicate the year)

3. My current duty station is ... (name of institution)

   in ... (name of city or district)

4. The highest degree I obtained is that of ...

5. Other degrees that I obtained are the following: ...

6. Number of years of professional experience in the health sector: ... (indicate number)

7. I was born in the year 19...

8. I am a O Male
   O Female

9. I declare to have completed this survey to the best of my ability: yes / no

10. I declare to have completed this survey on my own, without anyone’s assistance: yes / no

11. I would be interested to receive a summary of the findings of this survey: yes / no

*As researchers we are planning to conduct a series of focus group discussions around the following three topics: (a) drivers of health sector performance in Rwanda, (b) shortfalls in maternal health, and (c) possibilities to further strengthen the health system in Rwanda so as to improve maternal health.*

12. I would be interested to participate in a focus group discussion: yes / no

13. I have the following comments, suggestions for your group of researchers: ......

***You have reached the end of the survey.***

**MERCI BEAUCOUP !** **MURAKOZE CHANE !** **THANK YOU VERY MUCH !**
